# The activity of Nef on HIV-1 infectivity

**DOI:** 10.3389/fmicb.2014.00232

**Published:** 2014-05-20

**Authors:** Stéphane Basmaciogullari, Massimo Pizzato

**Affiliations:** ^1^Hôpital Necker-Enfants Malades, Sorbonne Paris Cité, Université Paris DescartesParis, France; ^2^INSERM U845Paris, France; ^3^Centre for Integrative Biology, University of TrentoTrento, Italy

**Keywords:** HIV, AIDS, auxiliary proteins, Nef, retrovirus infectivity

## Abstract

The replication and pathogenicity of lentiviruses is crucially modulated by “auxiliary proteins” which are expressed in addition to the canonical retroviral ORFs *gag, pol*, and *env*. Strategies to inhibit the activity of such proteins are often sought and proposed as possible additions to increase efficacy of the traditional antiretroviral therapy. This requires the acquisition of an in-depth knowledge of the molecular mechanisms underlying their function. The Nef auxiliary protein is expressed uniquely by primate lentiviruses and plays an important role in virus replication *in vivo* and in the onset of AIDS. Among its several activities Nef enhances the intrinsic infectivity of progeny virions through a mechanism which remains today enigmatic. Here we review the current knowledge surrounding such activity and we discuss its possible role in HIV biology.

## A BRIEF HISTORY OF THE DISCOVERY OF THE Nef ORF

The first reports of the entire HIV-1 genome sequences exposed an ORF, partially overlapping with the 3′LTR, which was initially named 3′ORF (Ratner et al., 1985; Sanchez-Pescador et al., 1985). Early studies demonstrated that its gene product was antigenic during the course of natural infection (Arya and Gallo, 1986; Franchini et al., 1986). However, it took several years before its role *in vivo* and its molecular functions began to be understood. Initial studies suggested that 3′ORF encoded for a GTPase (Guy et al., 1987), a finding soon dismissed as a possible consequence of sample contamination with bacterial GTPases (Backer et al., 1991). Subsequent reports suggested that 3′ORF was a negative factor (hence the name *nef*, still in use today), because its over-expression was found to attenuate viral transcription and HIV replication in cell culture (Luciw et al., 1987; Ahmad and Venkatesan, 1988; Niederman et al., 1989). These findings were soon contradicted by later reports (Hammes et al., 1989; Kim et al., 1989) which attributed the negative effect to the LTR sequences maintained in Nef-encoding vectors and interfering with HIV gene expression.

The first evidence demonstrating the requirement for an intact *nef* allele in the maintenance of high viral load and the timely development of immunodeficiency came from Rhesus macaques infected with a mutated strain of SIVmac_239_ lacking the Nef ORF (Kestler et al., 1991). Further evidence came from patients who contracted infection with Nef-deleted viruses and manifested long-lasting low level of virus replication and delayed onset of the disease (Deacon et al., 1995; Kirchhoff et al., 1995). A positive effect of Nef on HIV-1 replication was eventually confirmed *in vitro* using primary cell cultures and, to a lesser extent, in transformed cell lines (Terwilliger et al., 1991; de Ronde et al., 1992; Zazopoulos and Haseltine, 1993; Miller et al., 1994; Spina et al., 1994).

## OVERVIEW OF Nef ACTIVITIES

The *nef* gene is only present in the genomes of primate lentiviruses, i.e., HIV-1, HIV-2, and SIV. It is translated from a multiply spliced mRNA which generates a protein of 27–32 KDa highly expressed from the early stages of the infection process. Based on crystal (Lee et al., 1996; Arold et al., 1997; Grzesiek et al., 1997) and NMR (Grzesiek et al., 1996, 1997) structures, we know that Nef is made of a globular core domain flanked by a flexible N-terminal arm and a C-terminal disordered loop. Residues crucial for the interaction with different host factors are located in all three regions of the protein. Nef is myristoylated, which contributes to its association with membranes, together with a stretch of basic aminoacids close to the N-terminus (Bentham et al., 2006). Indeed, a significant fraction of Nef is observed in association with the plasma membrane and perinuclear membrane complexes (Kohleisen et al., 1992; Fujii et al., 1996; Greenberg et al., 1997). Myristoylation may also contribute to prevent Nef from multimerizing (Breuer et al., 2006). The protein is also detected within virion particles (Pandori et al., 1996; Welker et al., 1996, 1998; Bukovsky et al., 1997), a feature which could depend on the ability of Nef to associate with cellular membranes. Packaged Nef has also been reported to undergo cleavage by the viral protease (Bukovsky et al., 1997; Chen et al., 1998). However, as discussed below, the meaning and the specificity of Nef packaging into virions remain unclear.

Perhaps the most remarkable feature of Nef is its multi-functionality. Nef does not contain enzymatic activity, but exerts several cellular functions resulting from its ability to interact with numerous host factors. The most characterized activities of Nef result from the ability of the protein to connect with the cellular vesicular trafficking machinery and to perturb cell signaling.

### MODULATION OF CELL-SURFACE MOLECULES EXPRESSION LEVELS

Nef interacts with several proteins implicated in intracellular trafficking and modulates cell surface expression of several molecules (Landi et al., 2011). Nef down-regulates CD4 (Garcia and Miller, 1991) by enhancing its uptake into the endosome–lysosome compartment (Aiken et al., 1994; Chowers et al., 1994; Rhee and Marsh, 1994; Schwartz et al., 1995a; Bresnahan et al., 1998; Craig et al., 1998; Piguet et al., 1998, 1999; Janvier et al., 2001; Faure et al., 2004), a function conserved and maintained throughout disease progression that increases both virus infectivity and replication, as discussed in Section “Potential Effect of Nef During Virus Biogenesis.”

Nef affects the trafficking of many other proteins, which favors virus replication in the host by hiding or protecting infected cells from immune surveillance and by promoting virus dissemination. Because these properties are not strictly related to the ability of Nef to increase virus infectivity, they are mentioned in this chapter but the underlying mechanism will not be discussed further.

The ability of Nef to prevent the elimination of infected cells by the immune system is an important feature that favors virus dissemination in the host. Nef down-regulates molecules of the major histocompatibility complex-I (MHC-I; Schwartz et al., 1996) through a still debated mechanism distinct from that involved in CD4 down-regulation (Piguet et al., 2000; Blagoveshchenskaya et al., 2002; Williams et al., 2002, 2005; Larsen et al., 2004; Roeth et al., 2004; Lubben et al., 2007; Noviello et al., 2008; Dikeakos et al., 2012). This protects infected cells against killing by cytotoxic T cells (Collins et al., 1998), and is maintained under strong selective pressure only during the acute phase of infection, when the host is still fully immunocompetent (Carl et al., 2001). Protection against cell lysis is further achieved by Nef-dependent FasL up-regulation which triggers apoptosis of bysander cytotoxic cells (Xu et al., 1997, 1999). Of note, HIV and SIV Nef also interfere with MHC-II functions by down-regulating MHC-II complexes and up-regulating the MHC-II-associated II invariant chain (Schindler et al., 2003).

Cell infection by HIV or SIV is also characterized by T-cell receptor (TCR) pathway dysfunction. Nef can down-regulate the TCR/CD3 complex (Bell et al., 1998; Schaefer et al., 2000; Munch et al., 2002), a property restricted to alleles derived from SIV isolates non-pathogenic to their natural host, which might explain the higher virulence of HIV compared with SIV (Schindler et al., 2006). In addition, TCR activity can also be inhibited by the Nef-dependent down-regulation of the co-stimulatory molecule CD28 (Bell et al., 2001; Swigut et al., 2001). Finally, SIV Nef down-regulates the restriction factor BST-2 and ensures efficient release of viral particles from infected cells (Jia et al., 2009).

Key to these activities of Nef is the ability to form ternary complexes with cargo molecules and adaptor or coatamer complexes via a ExxxLL acidic di-leucin motif (Aiken et al., 1994) a EE di-acidic sequence located in its C-terminal loop (Piguet et al., 1999), a EEEE acidic cluster (Piguet et al., 2000) and a YxxΦ motif (where Φ represents a hydrophobic residue) on its N-terminal arm (Lock et al., 1999).

### MODULATION OF T-CELL ACTIVATION

In addition to modulating receptor expression levels, Nef also hijacks signaling pathways and alters the activation threshold of lymphocytes (Baur et al., 1994; Alexander et al., 1997; Schrager and Marsh, 1999; Simmons et al., 2001) by interacting with Src family tyrosine kinases (Saksela et al., 1995), members of the p21-activated serine/threonine kinases (Sawai et al., 1994, 1996; Khan et al., 1998; Renkema et al., 1999; Agopian et al., 2006) and Vav (Fackler et al., 1999; Rauch et al., 2008). This leads to a transcriptional program resembling that triggered by TCR stimulation, which might create a favorable intracellular milieu for virus replication. Signaling perturbation by Nef also results in the inactivation of cofilin, which inhibits cytoskeleton rearrangement and cell motility (Stolp et al., 2009). Features of the Nef protein, which are reported to contribute to this activity, include a PxxP proline-rich motif (Saksela et al., 1995), an amphipathic α-helix in the N-terminal arm (Baur et al., 1997) and a hydrophobic surface within the C-terminal loop (Agopian et al., 2006). Given that Nef increases cell-free virus infectivity in non-T-cell systems, the effect on primary T-cell activation does not seem to correlate with the activity of Nef on virus infectivity.

### THE EFFECT OF Nef ON RETROVIRUS INFECTIVITY

With the term infectivity we here indicate the efficiency with which the virus establishes an infection event within a cell, which culminates with the integration of the virus genome into the host cell genome. This is therefore a parameter which does not depend on steps of the virus life cycle which follow integration, such as virus gene expression or virus release. Infectivity is measured by relating the number or the frequency of the infectious events produced by cell-free virus with the physical number of virus particles. To compare infectivity across different samples, infectious events are therefore normalized to the physical virus content in the inoculum, determined by quantifying the amount of p24 CA protein or the RT-activity of the virus. Most studies investigating HIV-1 infectivity employ infection assays limited to a single round of viral replication by using trans-complemented molecular clones or by the addition of AZT or entry inhibitors at various time points following infection with replication competent viruses, in order to avoid the contributions from successive rounds of replication to the overall phenotype. Steps of the virus life cycle where the effect of Nef on infectivity can be manifest include receptor interaction, entry, uncoating, reverse transcription, nuclear import and integration.

The Guatelli lab was the first to report that HIV-1 lacking the ability to express Nef has lower infectivity compared with the Nef-positive counterpart (Chowers et al., 1994). This observation has then been confirmed by several labs using a variety of experimental systems differing for producer cell type, target cells and viral molecular clones (Aiken and Trono, 1995; Goldsmith et al., 1995; Miller et al., 1995; Tokunaga et al., 1998; Khan et al., 2001; Tobiume et al., 2001; Papkalla et al., 2002). Altogether, the magnitude by which Nef alters HIV-1 infectivity is highly variable, ranging from 3 to 40. In particular, the infectivity of Nef-defective HIV-1 seems to be the most impaired when virus is produced from lymphoid cell lines (Pizzato, 2010).

Additional observations indicate that the effect of Nef on infectivity plays a major function in the biology of primate lentiviruses. First, a comprehensive analysis including *nef* alleles derived from a large panel of diverse HIV and SIV isolates has demonstrated that the activity on infectivity is phylogenetically highly conserved (Munch et al., 2007). Second, functional analysis of *nef* alleles obtained during different stages of HIV infection revealed that the Nef effect on infectivity is maintained by a strong selective pressure during disease progression (Carl et al., 2001). Although this evidence suggests an important function for the Nef effect on infectivity, its precise role during the pathogenic infection remains to be deciphered. While an increased virus infectivity would logically imply an advantage on virus replication and therefore on viral load, clear evidence demonstrating such a link remains elusive.

### THE EFFECT OF Nef ON VIRUS REPLICATION

A positive effect of Nef on virus replication was first observed more than 20 years ago (Kim et al., 1989) and remains today mechanistically unclear. While the role of Nef for efficient virus replication *in vivo* is evident, this is not always the case in spreading infection *in vitro*, for which the effect of Nef is highly variable depending on the experimental system. The most robust requirement for Nef was observed using primary T-cells or macrophages infected before mitogenic stimulation of the cultures (Miller et al., 1994; Spina et al., 1994). An important contribution to this effect could therefore stem from the ability of Nef to alter T-cell activation status and favor preliminary virus replication before massive stimulation following exposure to mitogens.

In contrast, Nef seems to only have a modest effect on the replication of HIV-1 in transformed cell lines and in activated primary human T-cells. Discrepancy is observed between the marked effect of Nef on the infectivity of single round infection competent viruses and the modest Nef requirement for virus replication in the same cell cultures (Haller et al., 2011). One major difference between single round infections using cell-free virus and spreading infection of HIV throughout a cell culture is that in the latter cell-associated virus can be transmitted directly from cell to cell (cell-to-cell transfer; Jolly et al., 2004) which seems to be remarkably efficient [up to 1000-fold more efficient than cell-free virus (Sourisseau et al., 2007)]. A recent report indicates that Nef exerts only a modest positive effect on cell-to-cell transfer using both transformed cell lines and activated primary cells (Malbec et al., 2013), therefore overriding bigger differences from the contribution of cell-free virus (Haller et al., 2011). The role of the effect of Nef on infectivity on virus replication remains therefore to be elucidated.

## THE MECHANISTIC DETAILS OF THE EFFECT OF Nef ON INFECTIVITY

**FIGURE 1:**
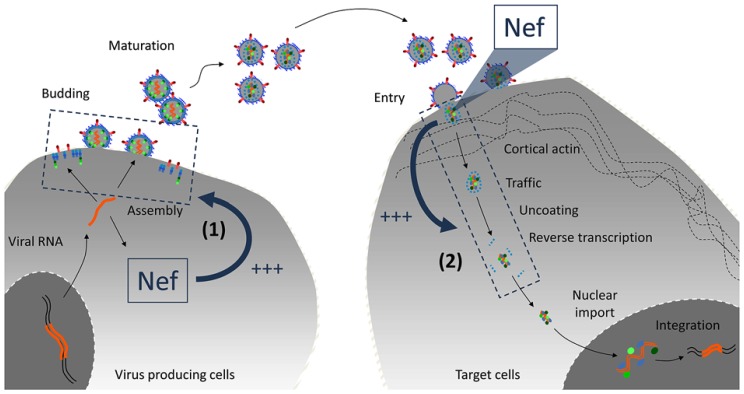
**Cellular or virus-borne: localization of Nef activity on virus infectivity.** Nef is expressed in infected cells and incorporated into virions. Given the profound imprint of Nef on the biology of infected cells, it may regulate a cellular function in virus-producing cells that favors virus infectivity (1). The fact that virus-borne Nef molecules are processed during maturation also suggests that cleaved molecules may play a role when virions hit target cells (2). Recent findings tend to favor the former hypothesis.

The effect of Nef on virus infectivity requires its expression in producer cells rather than target cells (Aiken and Trono, 1995). Nef might thus play a role as a virus-borne protein when virions hit target cells. Alternatively, in the presence of Nef, progeny virus particles might inherit a modification which is required to maintain their full infectious potential. Two sides of the same coin should therefore be considered. (1) What is the nature of the Nef-dependent modification inherited by the virus particle and how is it acquired? (2) In which step of the virus life cycle (ranging from receptor interaction to integration) is the infection of a target cell affected by such Nef-dependent modification? (**Figure 1**).

### INFECTIVITY: IS VIRUS-BORNE Nef DOING IT ITSELF?

Conflicting results have been published regarding the effect of Nef on fusion/entry which will be discussed later (Zhou and Aiken, 2001; Tobiume et al., 2003; Cavrois et al., 2004). Nevertheless the literature agrees that Nef+ viruses complete post-entry steps more efficiently than their Nef- counterparts (Aiken and Trono, 1995; Chowers et al., 1995; Schwartz et al., 1995b).The fact that Nef is incorporated into viral particles and cleaved by HIV-1 protease at residues W_57_L_58_ in the course of virion maturation makes it reasonable to hypothesize a specific role for virus-borne Nef in early steps of viral replication (Pandori et al., 1996; Welker et al., 1996; Bukovsky et al., 1997). Site-directed mutagenesis led to the identification of molecular species with a range of phenotype regarding their incorporation and maturation in virions; however, no correlation could be drawn between incorporation/maturation and infectivity because mutants were also deficient in other known functions of Nef, mostly CD4 or MHC down-regulation (Bukovsky et al., 1997; Miller et al., 1997; Chen et al., 1998; Welker et al., 1998; Bentham et al., 2006). The fact that Nef can be also incorporated into MLV particles (without affecting virus infectivity) further suggests that it is passively and “unpurposely” incorporated into enveloped virions due to its association with cell membranes (Bukovsky et al., 1997). Two papers by Laguette et al. (2009a) and Qi and Aiken (2008) addressed the question of Nef incorporation and its correlation to infectivity by fusing WT Nef proteins with a “viral carrier protein”, Vpr and CypA, respectively. These results support the idea that a role of Nef in the course of virus biogenesis, not as a virus-borne factor, likely accounts for its effect on virus infectivity, and are in line with the inability of Nef to complement the infectivity of Nef- viruses when it is expressed in target cells (Aiken and Trono, 1995; Pizzato et al., 2008).

### POTENTIAL EFFECT OF Nef DURING VIRUS BIOGENESIS

One of the first effects attributed to Nef is its ability to down-regulate cell surface CD4 expression level in infected cells (Guy et al., 1987). Because CD4 is the primary receptor for HIV (Dalgleish et al., 1984; Klatzmann et al., 1984), its down-regulation from the plasma membrane spares cells from cytotoxic super-infection and favors virus dissemination (Benson et al., 1993; Little et al., 1994). Furthermore CD4 down-regulation prevents the formation of CD4/gp120 complexes in intracellular compartments and at the plasma membrane, which has been shown to interfere with envelope glycoproteins (Env) incorporation into nascent virions and to decrease virus infectivity (Lama et al., 1999; Cortes et al., 2002; Arganaraz et al., 2003; Lundquist et al., 2004; Schiavoni et al., 2004). This phenotype appears to be of particular importance for primate lentiviruses since HIV and SIV Nef alleles, but also Vpu and Env, down-regulate cell surface CD4 through distinct mechanisms (Garcia and Miller, 1991; Benson et al., 1993; Sanfridson et al., 1994; Chen et al., 1996; Fujita et al., 1997; Hua and Cullen, 1997; Margottin et al., 1998; Schubert et al., 1998; Wildum et al., 2006; Laguette et al., 2009b; Magadan et al., 2010). CD4 down-regulation by Nef thus seems to favor HIV infectivity and replication as correlated by the Aiken and Kirchhoff groups (Glushakova et al., 2001; Lundquist et al., 2002). However, the sole ability of Nef to down-regulate CD4 cannot explain its effect on virus infectivity since Nef remains capable of increasing virus infectivity when virions are produced from CD4-negative cells or when virions are pseudotyped with the MLV-A envelope glycoprotein that does not interact with CD4 (Aiken and Trono, 1995; Miller et al., 1995; Aiken, 1997; Pizzato et al., 2008). In addition, Nef does not seem to modulate Env incorporation into the viral membrane when virions are produced from CD4-negative cells (Miller et al., 1995; Lai et al., 2011). The effect on infectivity is therefore an independent activity of Nef.

The hypothesis of a role of Nef in the course of virus biogenesis was further investigated in a comparative proteomic analysis of WT and Nef-defective NL4-3 viral particles intended to identify differences in their composition that might explain the higher infectivity of WT viruses (Bregnard et al., 2013). This study revealed that Ezrin and EHD4 are more abundant in Nef-defective than in WT NL4-3 virions but failed to demonstrate a direct inhibitory effect of these virus-borne proteins on Nef-defective virus infectivity. On the contrary, Ezrin and EHD4 depletion decreased WT viruses infectivity but not that of Nef-defective viruses, which supports their roles as possible co-factors in the Nef-mediated increase of virus infectivity. A similar comparative analysis has been conducted to identify a specific signature of Nef on the lipid composition of the viral membrane (Brugger et al., 2007). Although differences could be identified between Nef+ and Nef- derived membranes, none was found to account for the Nef-dependent increase of virus infectivity. Nef-dependent post-translational modification of viral proteins could also account for the higher infectivity of Nef+ over Nef- viruses. Although Nef-associated kinases were found to induce matrix phosphorylation on serine residues (Swingler et al., 1997), matrix was later found dispensable not only for virus replication, but also for Nef responsiveness, which ruled out its contribution to the phenotype (Reil et al., 1998; Dorfman et al., 2002). Of note, matrix deletion from Gag requires mutation or deletion of HIV-1 Env cytoplasmic tail to ensure the incorporation of the retroviral glycoprotein into the viral membrane and the biogenesis of infectious virions (Ono et al., 1997; Reil et al., 1998; Murakami and Freed, 2000; Ono et al., 2000; Tedbury et al., 2013). Simultaneous mutations might thus interfere and hide the requirement for matrix phosphorylation in the Nef-dependent increase of virus infectivity. Further investigation is thus required to formally identify Nef-dependent modifications of viral proteins, or incorporation/exclusion of cellular factors into/from virions, that might directly affect virus infectivity.

### EFFECT OF Nef (OR MODIFICATIONS INHERITED FROM PRODUCER CELLS) IN TARGET CELLS

Although Nef does not seem to affect the concentration of Env on the virus surface, its ability to increase virus infectivity is somewhat dependent on the mechanism that promotes fusion between the virus and the cell membrane and was long thought to be linked to the route of viral entry. While Nef increases the infectivity of viruses decorated with Env that allow for fusion at neutral pH such as that of HIV and A-MLV (referred to as responsive Env), no effect of Nef is observed with Env that requires virus endocytosis and endosome acidification to promote fusion such as that of VSV and RSV-A (Miller et al., 1995; Aiken, 1997; Luo et al., 1998; Pizzato et al., 2008). This has suggested the existence of a post-entry block encountered by incoming capsids when fusion takes place at the plasma membrane and counteracted by Nef (or its effects inherited from the virus producing cell, see below) or bypassed by incoming virions traveling through the endocytic network, out of reach of cytoplasmic factors. The cortical actin network has been suggested to account for such post-entry block, based on the relieving effect of actin-targeting drugs on the poorly infectious Nef- viruses but not on the fully infectious Nef+ viruses (Campbell et al., 2004). However, although Nef increases the infectivity of HIV-1, regardless of its tropism for CXCR4 or CCR5 receptors, not all HIV-1 Envs are equally responsive to Nef (Chazal et al., 2001; Papkalla et al., 2002; Lai et al., 2011; Usami and Gottlinger, 2013). Furthermore, the correlation between Nef responsiveness and entry at the plasma membrane is at odds with evidence of HIV entry into target cells in early endosomes, after endocytic vesicles have passed the cortical actin network but before endosome acidification (Miyauchi et al., 2009; van Wilgenburg et al., 2014). In addition, Nef responsiveness can be observed even when fusion takes place in acidic endosomes, in experimental setting where cells express RSV-A Env and virions are pseudotyped with the cognate Tva receptor (Pizzato et al., 2008).

Nef might thus have an effect on fusion that correlates neither with the route of entry, nor with Env dependence on pH. Such effect has been investigated with responsive and non-responsive Envs and led to contradictory results. While a Nef-dependent increase of the fusion process extrapolated from virus/cell or intravirion fusion assays has been reported by some groups (Schaeffer et al., 2001; Zhou and Aiken, 2001), others have failed to detect any difference between Nef+ and Nef- viruses in the completion of the fusion step, including groups that used the most quantitative βlam-Vpr fusion assay developed in the Greene laboratory (Miller et al., 1995; Tobiume et al., 2003; Campbell et al., 2004; Cavrois et al., 2004; Basmaciogullari, personal communication). Such conflicting results led Cavrois et al. (2004) to cautiously interpret the sensitivity of the βlam-Vpr assay and the hypothetical effect of Nef on fusion. The authors suggested that Nef might assist the enlargement of the pore arising from the fusion between the virus and cell membranes, and promote translocation of the viral capsid into the cell cytoplasm. Small pores arising from the fusion between Nef- viruses and cell membranes would restrict capsid translocation but allow for full diffusion of the fluorogenic substrate or βlam-Vpr. This would explain why Nef- viruses are less infectious than Nef+ viruses yet induce identical βlam-Vpr readouts. However, this model is based on the assumption that pore size does not limit substrate or βlam-Vpr diffusion, which needs to be demonstrated. Alternatively, if small pores restrict diffusion, hence βlam-Vpr positivity, identical βlam-Vpr readouts obtained with Nef+ and Nef- viruses can only be achieved if large pores are underrepresented and have a negligible contribution to the overall βlam-Vpr signal (i.e. Nef+ viruses: 99% small pores and 1% large pores/full entry; Nef- viruses, 99.9% small pores and 0.1% large pores/full entry). The fact that fusion inhibitors clearly decrease infectivity and the Vpr-βlam signal does not support this hypothesis. It thus seems that the most likely explanation for identical βlam-Vpr readouts is that Nef has no effect on fusion/capsid delivery but affects post-fusion steps of the virus life cycle. Of Note, Day et al. (2004), who also used this assay, reported a fusion advantage of Nef+ viruses over Nef- viruses, which highlights the need for a more robust and sensitive fusion assay in order to clarify the possible effect of Nef on fusion.

Besides these discrepancies on fusion, it has been shown that Nef affects the accessibility of neutralizing antibodies directed against the MPER region of gp41 in a cell/virus fusion context (Lai et al., 2011). Although this did not fully correlate with the ability of Nef to increase virus infectivity, it nevertheless demonstrates that Nef might affect Env proteins conformation or the lipid environment adjacent to the MPER region and thus the fusion capacity of Env glycoproteins. The difference between responsive and non-responsive HIV-1 Env was recently mapped to an epitope within the V2 region of gp120 (Usami and Gottlinger, 2013). Yet, given the divergence between Nef responsive HIV-1 and MLV-A Env glycoprotein sequences and the responsiveness of Tva-pseudotyped viruses (Pizzato et al., 2008), the common parameter that allows for Nef responsiveness remains unknown. It thus seems that proteins found on the virus surface that mediate fusion, whether they be viral Envs or cognate receptor(s), are major determinants of Nef responsiveness but most likely not through their role in fusion.

The effect of Nef has also been documented at the level of cDNA synthesis in target cells. Although early experiments could not discriminate between effects of Nef on fusion or post-fusion steps, they nevertheless demonstrated that Nef+ viruses generate more early reverse transcription products than Nef- viruses, supporting an effect of Nef operating anywhere between fusion and viral DNA translocation to the nucleus (Aiken and Trono, 1995; Schwartz et al., 1995b). Of note, intravirion stimulation of reverse transcription was shown to compensate for the effect of Nef on virus infectivity (Khan et al., 2001). Given the interdependence between uncoating and RT, these results suggest that Nef might assist either of the mechanisms (Hulme et al., 2011; Yang et al., 2013). Although Nef does not show any effect on uncoating *in vitro*, an effect of Nef *in vivo* cannot be ruled out (Forshey and Aiken, 2003).

### CONVERGING MECHANISMS

Three papers published recently describe a striking parallel between HIV-1 Nef and MLV glycoGag (Pizzato, 2010; Usami and Gottlinger, 2013; Usami et al., 2014). This protein arises from the translation of the unspliced MLV RNA from a CUG initiation codon upstream from the conventional initiation codon of Gag, which results in the addition of 88 residues in frame and N terminal to Gag, responsible for the type II orientation of the corresponding protein where the added N terminal residues constitute the transmembrane domain and extend into the cytoplasm of the cell. It has been shown that ectopic expression of Nef or glycoGag similarly increases the infectivity of viruses produced from cells transfected with a Nef- provirus. Interestingly, simultaneous co-expression of Nef and glycoGag has no synergistic effect and glycoGag has no effect on VSV-G pseudotyped viruses, suggesting that these proteins are involved in the same mechanism that eventually leads to the increase of virus infectivity (Pizzato, 2010). In addition the V2 region of HIV-1 Env, which dictates Nef responsiveness, also dictates responsiveness to glycoGag (Usami and Gottlinger, 2013). Finally, although the ability of Nef to increase virus infectivity and its effect on the accessibility of MPER epitopes on HIV-1 Env partially overlap, glycoGag expression also affects the accessibility of similar MPER epitopes (Lai et al., 2011). This further confirms the converging functions of these unrelated proteins.

## MECHANISTIC HYPOTHESES

### THE BROAD PICTURE

**FIGURE 2:**
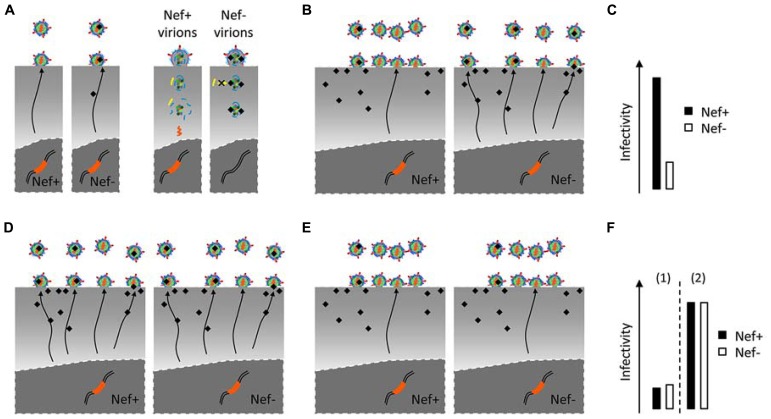
**Possible mechanisms responsible for the differential infectivity of Nef+ and Nef- viruses. (A)**, Nef- virions may acquire a defect during biogenesis which could be either the packaging of an inhibitor into virions (black shape), or the exclusion of a cofactor (not shown). This defect might then prevent the recruitment of cofactor (yellow shape) or be the target of an inhibitor (not shown) in target cells. **(B,C)** The effect of Nef on virus infectivity is evident when virus is produced from Nef-responsive cells, in which Nef regulates such inhibitor or promote virus assembly to subcellular locations where the defect is not acquired **(B)**. **(D–F)** In contrast, Nef-non-responsive producer cells generate Nef+ and Nef- viruses with similar infectivities **(F)**. Two possibilities may explain this phenotype: Nef fails to protect the virus from the defect [**D,F(1)**, Virions have suboptimal infectivity even in the presence of Nef]; alternatively, producer cells lack the cause of the defect or target virus assembly away from inhibitors [**E,F(2)**, Nef- virus already has optimal infectivity].

Several hypotheses can be put forward in a two-act scenario responsible for the differential infectivity of Nef+ and Nef- viruses. Nef might hijack cellular pathways in virus-producing cells that ultimately optimize infectivity (modification of viral proteins or viral content beyond its known elements). Cellular pathways in target cells might then specifically assist Nef+ viruses in the early steps of the virus life cycle (**Figures 2A,C**). Both sides of this same coin can then either involve cofactors recruited by Nef or inhibitory factors counteracted by Nef. So far, the lentiviral auxiliary proteins Vif, VpU, and VpX have been involved in the neutralization of an inhibitory factor (or restriction factor) such as Apobec3, Bst2, and SAMHD1. In the case of Nef both possibilities remain open. The Nef requirement for reaching optimal infectivity is highly variable and depends on the cell types from which virions are produced (Pizzato, 2010), it is thus plausible that such variability is conferred by differential expression of one or more cellular genes involved in the scenario depicted above.

### WHAT IS Nef DOING TO THE VIRUS, HOW AND WHERE?

Comparative analysis of Nef+ and Nef- viral particles have clearly revealed differences in the lipid and protein composition, confirming that Nef expression in virus-producing cells has an impact on the virus biogenesis. Although the modification of the viral lipid bilayer by Nef did not seem to account for the higher infectivity of Nef+ viruses over Nef- viruses, further investigation with more sensitive fusion assays that are yet to be developed might reveal a direct link between the viral membrane lipid composition, membrane fusion and infectivity. Differences in the protein composition of viral particles also revealed partial depletion or enrichment in particular proteins, depending on the expression of Nef in virus-producing cells. Ezrin and EHD4 were found in higher concentration in Nef- viruses and their involvement in the infectivity phenotype confirmed; however, their relative excess did not seem to account for the poor infectivity of Nef- viruses. Rather, it was interpreted that Ezrin and EHD4 are hijacked by Nef in the process of increasing virus infectivity, thus preventing their passive incorporation into virions.

It is interesting to note the converging phenotype of the *nef*- mutation and mutations of p24CA on serine residues: both mutants are poorly infectious but their infectivity is restored by VSV-G pseudotyping (Brun et al., 2008). Although this is far from explaining how Nef increases virus infectivity, it nevertheless confirms that post-translational modification by Nef is an interesting line of investigation to follow. Additional work is thus required in order to identify the differences between Nef+ and Nef- responsible for the differential behavior of viruses when they hit target cells.

As presented in this review, a main functional feature which characterizes Nef alleles and is required for several Nef activities, is the ability to intersect with the vesicular trafficking machinery of the cell. As we discussed earlier, Nef is capable of interacting with AP1, AP2, and βCOP1 via distinct and discrete motifs present in HIV and SIV Nef molecules (ExxxLL, EE, EEEE, YxxL). Many observations converge toward a fundamental role of vesicular trafficking for the activity of Nef for infectivity. In particular: (1) HIV-1 Nef requires the integrity of the AP2 interacting motif ExxxLL in virus producing cells (Chowers et al., 1994). (2) Similarly, SIV Nef requires the YxxL motif (Lock et al., 1999). (3) Mutant HIV-1 Nef proteins, which do not interact with dynamin 2 (crucial for intracellular vesicles biogenesis), are no longer capable of increasing HIV infectivity (Pizzato et al., 2007). (4) The activity of Nef on infectivity requires functional clathrin-mediated endocytosis, because it is impaired by silencing clathrin gene expression and by over-expressing transdominant-negative dynamin 2 and AP180. (Pizzato et al., 2007). (5) Glycogag requires and interacts with AP2 via a YxxL motif to rescue the infectivity of Nef-defective HIV-1 (Usami et al., 2014). 5) EHD4 and Ezrin silencing render Nef+ viruses as poorly infectious as Nef- viruses (Bregnard et al., 2013).

As highlighted earlier, doubts remain on the possibility that Nef affects cytoplasmic delivery of HIV. In the hypothesis where Nef enhances the efficiency of fusion between the virus and the cell membranes, it might do so by promoting the endocytosis of a membrane-bound fusion inhibitor in virus producing cells otherwise incorporated into viral membrane. More generally, factors with potential inhibitory effect, or responsible for the recruitment of co-factors in target cells, might also be rerouted away from or to the viral assembly platforms through the deregulation of protein trafficking by Nef in infected cells.

Another interesting challenge is the identification of the cellular compartment where Nef+ virions acquire their phenotype. Nef might drive the viral components to the appropriate assembly platforms where virions acquire specific features responsible for their higher infectivity and, as suggested earlier, the nature of the protein that decorates virions might play a role in this sorting mechanism, independently of their involvement in membrane fusion. Despite some flexibility in virus pseudotyping, much evidence suggests that the acquisition of Env by budding virions is somewhat regulated (Johnson et al., 1998; Jorgenson et al., 2009; Muranyi et al., 2013; Roy et al., 2013). Env might be targeted by Nef and rerouted to ensure assembly at specific sites (**Figure 2B**). Of note, similar infectivities of Nef– and Nef+ viruses might either reflect the inability of Nef to drive virus assembly to such compartments (**Figures 2D,F**) or indicate that optimal assembly does not require the presence of Nef (**Figures 2E,F**). Because cytoplasmic tail-deleted Envs are still Nef responsive, specific determinants might lie in the extracellular domain, and interact with a chaperone along its secretion and delivery to the plasma membrane. Although this awaits formal evidence, it is compatible with the recent identification of a V2 region in HIV-1 Env as a major determinant of responsiveness to Nef/glycoGag.

### CONSEQUENCES IN TARGET CELLS

Tracking virions in target cells is particularly challenging, especially when virions cannot be pseudotyped with VSV-G. For this reason, not much is known about possible functions of virus-borne molecules in early steps of the life cycle. Whether Nef itself or the modifications it brings to viral particles impact on the completion of early steps, this most likely relies on the recruitment of co-factors or the neutralization of inhibitory factors in target cells. A cutting edge genetic approach based on the screening of a human siRNA library has allowed for the identification of cellular factors that modulate HIV-1 replication (HDFs, HIV-1 dependency factors). However, virions used in such HTS were either Nef- or VSV-G pseudotyped, excluding *de facto* the possibility to identify HDFs involved in the ability of Nef to increase virus infectivity (Brass et al., 2008; Konig et al., 2008; Zhou et al., 2008). A similar screening performed in experimental settings where Nef+ and Nef- viruses could be compared such as that described by Bregnard et al. (2013), would shed light on the pathways usurped by Nef to increase virus infectivity.

## CONCLUSION

Biochemistry-based approaches have been used in order to identify cellular factors involved in the many functions of Nef. Affinity tagging or immunoprecipitation followed by mass spectrometry (Pizzato et al., 2007; Jager et al., 2012; Mukerji et al., 2012) and conventional or ubiquitin-split yeast two-hybrid screening (Benichou et al., 1997; Kammula et al., 2012) have identified Nef binding partners. Omics methods have also been used to identify how HIV infection or Nef expression alone modifies the biology of the cell (Schrager and Marsh, 1999; Wang et al., 2000; Simmons et al., 2001; Kramer-Hammerle et al., 2005; van’t Wout et al., 2005; Berro et al., 2007; Ringrose et al., 2008; Kramer et al., 2012). Given the many Nef partners and cellular processes affected by Nef identified so far, a strategy focused on the analysis of viral particles might better address the question of the effect of Nef on virus infectivity. Mass-spectrometry based techniques and computing tools have strikingly improved, which makes possible the analysis of Nef-induced post-translational modification of HIV-1 proteins in order to reveal specific differences between Nef+ and Nef- virions. In addition, due to the convergence of Nef and glycoGag on the infectivity phenotype, comparing the results of experiments carried out with these proteins might narrow down the list of modifications relevant for virus infectivity.

## Conflict of Interest Statement

The authors declare that the research was conducted in the absence of any commercial or financial relationships that could be construed as a potential conflict of interest.
